# Infectious complications in patients undergoing transrectal prostate-biopsy with ciprofloxacin compared to fosfomycin-trometamol

**DOI:** 10.1007/s00345-025-05818-8

**Published:** 2025-07-26

**Authors:** Andreas Banner, Sebastian Ubber, Ursula Stoces, Christine Meyer, Stephan Madersbacher, Igor Grabovac

**Affiliations:** 1 Department of Urology, Klinik Favoriten, Kundratstrasse 3, Vienna, A-1100 Austria; 2 Department of Social and Preventive Medicine, Centre for Public Health, Medical University of Vienna, Vienna, Austria; 3 Sigmund-Freud Private University, Vienna, Austria

**Keywords:** Prostate cancer, Prostate biopsy, Infection, Complication, Prevention

## Abstract

**Purpose:**

To evaluate the risk of infectious complications associated with the use of ciprofloxacin (CIP) or fosfomycin-trometamol (FMT) for antibiotic prophylaxis in patients undergoing transrectal prostate biopsy (TrBx).

**Methods:**

A retrospective, single-centre analysis was conducted, including patients who underwent TrBx from 2015 to 2023 and received CIP or FMT for antimicrobial prophylaxis. The primary endpoint was symptomatic urinary tract infections within 30 days after TrBx, and the secondary endpoint was hospitalization due to infectious complications. Logistic regression was used to assess the risk of infection, adjusted for variables such as age, prostate size and comorbidities. Hospitalization rates were compared using the chi-square test.

**Results:**

Overall, 913 patients were eligible for inclusion, of whom 491 had received CIP and 422 had received FMT. Infectious complications occurred in 38/913 (4.2%) of all patients, 12/491 (2.4%) of whom had received CIP and 26/422 (6.2%) of whom had received FMT. Multivariable analysis revealed a significantly greater risk of infectious complications with FMT (adjusted odds ratio 2.99, 95% CI 1.39–7.16, *p* = 0.008) compared to CIP. Hospitalization rates were similar in the CIP group (8/12, 66.6%) and FMT group (14/26, 53.5%, *p* = 0.7), but one death occurred due to fulminant sepsis in the FMT group.

**Conclusion:**

Compared with CIP, FMT prophylaxis is associated with a greater risk of infectious complications and should be used cautiously in routine clinical practice. Given the uncertainty of FMT’s efficacy in preventing infections after TrBx, alternative antibiotic regimens should be preferred, or transitioning to the transperineal biopsy approach may further reduce infection risk and support antibiotic stewardship.

**Supplementary Information:**

The online version contains supplementary material available at 10.1007/s00345-025-05818-8.

## Background

Prostate cancer (PC) is the most common solitary tumor among men in Europe, with an estimated incidence rate of 473.3/100,000 in 2020 [1]. Diagnosis is primarily confirmed through prostate biopsy with approximately 1 Mio. procedures performed annually in the Medicare population in the U.S [2]. Prostate biopsies should ideally be performed using a multiparametric magnetic resonance imaging (mpMRI)-targeted and transrectal ultrasound (TRUS)-guided approach, whereas the biopsy route can be either transrectal (TrBx) or transperineal (TpBx) [3]. The European Association of Urology (EAU) guidelines recommend the transperineal route as the first choice when feasible, as a meta-analysis revealed lower rates of infectious complications with TpBx (3.3%) compared to TrBx (5.6%) [3, 4]. However, a 2022 survey among Austrian urologists revealed that more than 80% of participating departments still use TrBx as the standard approach, as the transition to TpBx presents challenges related to tolerability, technical feasibility and logistic issues [unpublished].

Preparation for TrBx should routinely include rectal povidone-iodine cleansing before biopsy in addition to antimicrobial prophylaxis, as meta-analyses have demonstrated reduced rates of infectious complications with this approach [4, 5]. Traditionally, fluoroquinolones (FQs) have been used for antimicrobial prophylaxis in this setting, but a recent increase in FQ resistance and regulatory changes by the European Medicines Agency (EMA) resulted in a suspension of indications for perioperative antimicrobial prophylaxis [6, 7]. Alternatives include antimicrobial prophylaxis on the basis of rectal swabs or stool cultures; augmented prophylaxis with antibiotic combinations; or alternative single agents such as cephalosporins, aminoglycosides or fosfomycin-trometamol (FMT) [5]. The latest EAU prostate cancer guidelines update revoked the recommendation for FMT as an antimicrobial agent before TrBx because of conflicting results from randomized controlled trials (RCTs) and nonrandomized controlled trials (nRCTs). A meta-analysis of RCTs indicated that FMT was superior to fluoroquinolones in preventing infectious complications. However, several limitations of these trials must be noted: [1] two of these trials had small sample sizes (268 and 300 patients); [2] different durations of antibiotic prophylaxis were used; and [3] baseline resistance screening was not performed. However, patients at elevated risk for multidrug-resistant pathogens were excluded, limiting transferability to real-world scenarios [4]. None of these studies reported on potential confounders of infection risk [5]. One study is only available in Spanish [5, 8–10]. On the other hand, a large Canadian nested case‒control study revealed a greater risk of urinary sepsis with FMT compared to ciprofloxacin (CIP) after adjusting for potential confounders [5, 11]. In all of these investigations, local resistance patterns must be considered when these results are interpreted, especially since the RCTs favouring FMT were conducted in regions with relatively high CIP resistance rates.

With the global transition to TpBx progressing slowly, and infectious complications after TrBx remaining a major drawback of prostate cancer screening, optimizing the periprocedural workflow with effective antimicrobial prophylaxis remains a critical objective [12, 13]. To further evaluate the risk of infectious complications in patients undergoing TrBx with FMT compared to those undergoing TrBx with CIP, we conducted a retrospective, single-center observational study.

## Patients and methods

We identified consecutive patients who underwent TrBx between 01.01.2015 and 31.12.2023 for suspected PCa or for active surveillance, in accordance with the recommendations of the EAU Guidelines [3]. Briefly, all patients underwent prostate-specific antigen (PSA) quantification, digital rectal examination (DRE) and mpMRI to estimate the risk of PC before biopsy. Two different regimens for antimicrobial prophylaxis were employed as standard operational procedures during the observation period, and patients who received other single-antibiotic or augmented prophylactic regimens were excluded. Eligible patients had a urological history with clinical examination findings, PSA levels (ng/ml), mpMRI results, TRUS findings and biopsy results extracted from our semiprospectively maintained database.

### Biopsy procedure and antimicrobial prophylaxis

All biopsies were performed by trained urologists following a standardized protocol. Prior to the procedure, all patients received counselling regarding the biopsy, including potential risks and benefits, and provided written informed consent. Patients were instructed to undergo rectal preparation using an enema and were advised to discontinue anticoagulants or antiplatelets in accordance with current clinical guidelines to minimize bleeding risk [14]. Preprocedural assessments and adjustments to medication were individualized on the basis of each patient’s medical history and risk factors. Screening for antimicrobial resistance was conducted using a rectal swab in patients with a history of antibiotic therapy in the last 6 months, a recent travel history to high-risk areas for multidrug-resistant pathogens, or a history of infection with multidrug-resistant pathogens or immunosuppression. Patients with confirmed resistance to the prescribed prophylactic antibiotic received either single-dose intravenous prophylaxis, alternative short-term oral prophylaxis, or an augmented regimen (intravenous, oral, or a combination) on the basis of microbiological findings and were therefore excluded from this investigation.

Patients were positioned in a left lateral position with their legs tucked upwards. The biopsies were conducted using the transrectal approach with the bk3000 and later the bkSpecto ultrasound systems (GE HealthCare), which were equipped with a high-resolution triplane transrectal transducer (E14C4t, GE HealthCare) and an automatic 18 Gauge disposable biopsy gun (Curaway Medical Technology Co., Ltd.). Following a periprostatic nerve block with 5 mL of 0.5% mepivacaine, all patients presenting with PI-RADS score ≥3 lesions on mpMRI underwent 10–12 core systematic biopsies with 12° needle guidance as well as with targeted saturation biopsies of suspicious lesions using technical TRUS-MRI fusion with the BioJet System (Medical Targeting Technologies GmbH) with 12° or 40° needle guidance, depending on the investigator’s choice. Since infectious complications were the primary focus of our investigation, patients with a PI-RADS score ≤2 or those without mpMRI who underwent systematic biopsy only were also included. Prostate imaging by mpMRI was permitted both onsite and offsite. All images of patients referred for prostate biopsy were reviewed, graded and mapped onsite by a single dedicated expert radiologist.

Periinterventional antimicrobial prophylaxis consisted of either three doses of oral ciprofloxacin 500 mg or two doses of oral fosfomycin-trometamol 3 g. For patients receiving CIP, the regimen consisted of one dose in the evening before the biopsy, the second dose in the morning of the biopsy day and the third in the evening of the biopsy day. For patients receiving FMT, the regimen consisted of one dose in the morning of the biopsy day and a second dose in the evening of the biopsy day.

The CIP regimen was first administered from 2015 to 2019 and then switched to the FMT regimen in accordance with the regulatory instructions from the EMA and recommendations from the EAU guidelines. Owing to an unexpectedly high infection rate in patients receiving the FMT regimen, our protocol was reverted to CIP in 2023.

Postprocedural history was retrospectively evaluated by reviewing our hospital reporting system. Patients were instructed to promptly report any postoperative complications, particularly clinical signs of urinary tract infections (UTIs) and/or fever. The majority of patients underwent routine follow-up at our center 10–14 days after the biopsy procedure to evaluate complications and review the biopsy results.

### Primary and secondary endpoints

The primary endpoint was defined as symptomatic UTI within 30 days of the biopsy procedure. All patients who presented to our outpatient clinic with suspected complications after TrBx routinely underwent a clinical examination; urine dipstick test; transabdominal ultrasound of the bladder, prostate and kidneys; tympanic temperature measurement; and blood sampling. Scrotal examination and ultrasound were only performed in suspected cases of epididymitis. Patients were instructed to retract their foreskin and disinfect the glans before collecting midstream urine in a sterile urine cup. A urine culture was obtained if abnormal findings were detected via the dipstick test. UTI was diagnosed on the basis of the following findings:

- Leukocyturia with dysuria, frequency, urgency, suprapubic or flank pain with or without subfebrile temperatures or fever ≥38.0 °C.
- Enlargement and hyperemia of the epididymis on ultrasound with or without abscess formation and with or without subfebrile temperatures or fever.
- Urosepsis was defined as an increase in the Sequential Organ Failure Assessment (SOFA) score of 2 points or more.

Patients suspected of having infections other than UTIs were further examined at the discretion of the treating physician. Notably, from 2020 to 2022, patients referred to our outpatient clinic underwent routine SARS-CoV-2 rapid antigen testing.

The secondary endpoint was defined as hospitalization due to infectious complications within 30 days of the biopsy procedure. Additionally, infectious complications were graded according to the Clavien‒Dindo classification (Supplementary Table 1) [15].

### Statistical analysis

We performed initial data analysis to identify missing or inconclusive values. As the percentage of missing data for each baseline characteristic was low (< 1%), missing values for continuous variables were imputed as the means of the overall cohort [16]. The categorical variables are reported as counts and percentages, whereas the continuous variables are reported as medians with interquartile ranges (IQRs) or means with standard deviations (SDs).

To mitigate confounding in the comparison of antibiotic groups (CIP vs. FMT), inverse probability weighting (IPW) was applied [17]. Propensity scores were estimated using a logistic regression model, incorporating key covariates to account for systematic differences between treatment groups. These scores were used to generate inverse probability weights, ensuring a balanced distribution of covariates across groups. Weighted analysis was subsequently conducted to provide an unbiased estimation of the associations between antibiotic choice and infectious complications.

Univariable and multivariable logistic regression models were fitted to estimate the relative risk of infectious complications, expressed as odds ratios (ORs) with 95% confidence intervals (95% CIs). The univariable model included the antibiotic group (CIP or FMT) as the independent variable. In the multivariable model, the risk of infectious complications was adjusted for clinically significant covariates, including age at biopsy, biopsy history, prostate size, and diabetes mellitus. We assessed the assumptions of logistic regression graphically, checking for heteroskedasticity and the linearity of the logit for each continuous predictor. Multicollinearity was evaluated using the variance inflation factor (VIF), and Cook’s distance was used to identify and exclude influential data points.

Several sensitivity analyses were conducted: First, patients who presented with infections characterized by nonspecific symptoms or who experienced prolonged latency before symptom onset were reclassified as non-cases. Second, a subset of patients was evaluated to assess the association between the total biopsy count per patient and infectious complications. Third, to explore potential confounding introduced by non-random resistance screening, an additional multivariable logistic-regression model that included resistance screening as additional covariate was fitted and compared to the primary model using analysis of variance (ANOVA).

Group differences in hospitalization rates due to infectious complications after prostate biopsy were analysed using the chi-square test.

All the statistical tests were conducted at a one-sided significance level of 0.05. All analyses were conducted using R version 4.4.1 (R Foundation for Statistical Computing, Vienna, Austria) with the packages broom (v1.0.6), dplyr (v1.1.4), gtsummary (v2.0.3) and tidyverse (v2.0.0) packages.

Institutional review board approval was obtained (EK 24-152-VK), and the investigations were conducted in accordance with the Declaration of Helsinki. Data protection was assured by storing data onsite in a password-secured file. Individual patient data were pseudonymized with continuous identification numbers prior to analysis. Access to the data was restricted to authorized doctors at the treating center only.

## Results

We identified 913 patients who underwent TrBx between 01.01.2015 and 31.12.2023 and were eligible for analysis. Antibiotic prophylaxis was CIP in 491 patients and FMT in 422 patients; the full consort diagram is shown in Supplementary Fig. 1. There were no significant differences in baseline characteristics between the two groups; only the rate of previous prostate biopsies was greater in patients in the CIP arm (38%) compared to patients in the FMT arm (19%) (Table 1). A full description of cases with infectious complications is provided in Table 2.

**Table 1:** Baseline characteristics, stratified by antibiotic prophylaxis

Characteristics^2^	Ciprofloxacin, *N* = 491 (53.8%)^1^	Fosfomycin, *N* = 422 (46.2%)^1^
Age (yrs)	68.0 (61.0–74.0)	67.0 (61.0–74.0)
PSA (ng/ml)	7.5 (5.6–11.0)	7.3 (5.2–10.8)
History of prostate biopsy	185 (37.7%)	82 (19.4%)
Prostate volume on MRI (ml)	46.0 (33.0–64.0)	41.0 (31.0–58.0)
Prostate volume on TRUS (ml)	45.0 (33.0–60.0)	40.0 (30.0–53.0)
DRE findings		
normal	382 (77.8%)	338 (80.1%)
abnormal	85 (17.3%)	84 (19.9%)
missing	24 (4.9%)	0 (0.0%)
PI-RADS score		
2	3 (0.6%)	6 (1.4%)
3	66 (13.4%)	30 (7.1%)
4	283 (57.6%)	249 (59.0%)
5	139 (28.3%)	137 (32.5%)
Rectal swab	103 (21.0%)	88 (20.9%)
Number of systematic cores	12.0 (10.0–12.0)	12.0 (10.0–12.0)
Number of targeted cores	11.0 (8.0–15.0)	9.0 (7.0–12.0)
Number of total cores	17.0 (11.0–20.0)	21.0 (18.0–23.0)
Cancer diagnosis	317 (64.6%)	327 (77.5%)

^1^Data are presented as n (%) or median (IQR)

^2^DRE digital rectal examination, IQR interquartile range, MRI magnetic resonance imaging, PSA prostate specific antigen, TRUS transrectal ultrasound

**Table 2:** Detailed description of infectious complications in patients who underwent transrectal prostate biopsy

ID	Antibiotic Prophylaxis	Symptoms	Onset after biopsy (d)	CDC-Grade	Admission	Therapy	Microbiologic samples	Susceptibility to prophylaxis	Comments
31	CIP	Fever, dysuria	2d	2	No	Antibiotics	Urine: n.a.	–	–
43*	CIP	Subfebrile, dysuria	2d	3a	7d	Antibiotics, fluids	Urine: n.a.	–	–
60	CIP	Fever, urgency	5d	2	3d	Antibiotics, fluids	Urine: n.a.	–	Suspicion of prostatitis on MRI
75	CIP	Fever, frequency	4d	2	No	Antibiotics	Urine: n.a.	–	–
125	CIP	Fever	1d	2	4d	Antibiotics, fluids	Urine: *E. coli*	No	–
171	CIP	Fever, urgency	4d	2	4d	Antibiotics, fluids	Urine: *E. coli*	Intermediate	–
184	CIP	Fever, frequency	10d	2	No	Antibiotics	Urine: neg.	–	–
241	CIP	Fever	1d	2	8d	Antibiotics, fluids	Urine: *E. coli 3MRGN*	No	–
342	CIP	Fever, suprapubic pain, retention	10d	3a	10d	Antibiotics, indwelling catheter	Urine: *E. coli 3MRGN*	No	–
358	CIP	Fever, dysuria, urgency	2d	2	5d	Antibiotics, fluids	Urine: *E. coli*	No	–
410	CIP	Fever, dysuria	3d	2	No	Antibiotics	Urine: *E. coli*	No	–
527	FMT	Fever, dysuria	n.a.	2	4d	Antibiotics, fluids, analgesics	Urine: *E. coli*	n.a.	Treated at external clinic
534	FMT	Fever, retention	2d	3a	3d	Antibiotics, fluids indwelling catheter	Urine: *K. oxytoca*	No	Patient was first treated ambulatory, admitted 12d later with recurrent fever
558	FMT	Fever, hematuria, malaise	3d	2	3d	Antibiotics, fluids	Urine: *E. coli*	n.a.	–
573	FMT	Fever, urgency	6d	2	Yes	Antibiotics, fluids	Urine: neg. Blood: *B. fragilis gr.*	No	Duration of admission unknown
595	CIP	Fever, retention, epididymitis	n.a.	3a	n.a.	Antibiotics, fluids, indwelling catheter	Urine: n.a.	–	Treated at external clinic, transferred during treatment
621	FMT	Fever, dysuria	9d	2	No	Antibiotics, analgesics	Urine: *E. coli*	n.a.	–
639	FMT	Fever, urgency, frequency	4d	2	4d	Antibiotics, fluids analgesics	Urine: n.a. Blood: n.a.	–	Clinical course with leukocytosis and elevated CRP (~ 250 mg/L) highly suspicious for bacterial infection
645	FMT	Fever, dysuria	1d	2	5d	Antibiotics, fluids analgesics	Urine: neg. Blood: neg	–	Antibiotics established before sampling
647*	FMT	Subfebrile, dysuria	10d	2	No	Antibiotics	Urine: neg.	–	–
649	FMT	Subfebrile, epididymitis	7d	2	No	Antibiotics, analgesics	Urine: n.a.	–	–
683	FMT	Fever, suprapubic pain	3d	2	No	Antibiotics, analgesics	Urine: n.a.	–	–
691	FMT	Fever, retention	1d	2	No	Antibiotics, analgesics	Urine: neg.	–	–
711	FMT	Fever, malaise	2d	2	No	Antibiotics	Urine: *E. coli*	Yes	–
751*	FMT	Fever, dysuria	15d	2	No	Antibiotics	Urine: *E. coli*	n.a.	–
763*	FMT	Fever, epididymitis	16d	2	No	Antibiotics, analgesics	Urine: neg.	–	Received indwelling catheter for retention before occurrence of epididymitis
797	FMT	Fever, malaise	4d	2	3d	Antibiotics, fluids	Urine: neg. Blood: neg.	–	Antibiotics established before microbiologic sampling
821	FMT	Fever, suprapubic pain	1d	2	No	Antibiotics	Urine: neg.	–	–
839	FMT	Fever, frequency	1d	2	3d	Antibiotics, fluids	Urine: neg.	–	–
850*	FMT	Hematuria, suprapubic pain	3d	2	No	Antibiotics	Urine: neg.	–	–
873*	FMT	Subfebrile epididymitis, retention	4d	3a	3d	Antibiotics, fluids	Urine: neg.	–	Received indwelling catheter for retention before occurrence of epididymitis
880	FMT	Fever	4d	2	5d	Antibiotics, fluids	Urine: *K. pneumonia*	Yes	–
907	FMT	Fever	2d	2	4d	Antibiotics, fluids	Urine: n.a. Blood: neg.	–	–
928	FMT	Fever, dysuria	2d	5	3d	Antibiotics, fluids, abscess drainage (prostate), ICU, ECMO	Urine: *K. pneumoniae*	Yes	Received outpatient treatment initially, showing a response to antibiotic therapy with symptom remission. After experiencing a recurrence of fever, the patient did not seek immediate follow–up care at the urology department After intensive care measures were exhausted, the cause of death was septic shock with multiorgan failure
931	FMT	Fever, urgency	1d	2	No	Antibiotics	Urine: neg.	–	–
942	FMT	Fever	4d	2	3d	Antibiotics, fluids	Urine: *E. coli*, *E. faecalis*	No	–
952	FMT	Epididymitis	4d	2	9d	Antibiotics, analgesics	Urine: neg.	–	–
957	FMT	Fever, dysuria, frequency, retention	9d	3a	No	Antibiotics, indwelling catheter	Urine: neg.	–	–

CDC Clavien-Dindo-Classification CIP Ciprofloxacin CRP C-reactive protein ECMO Extracorporeal membrane oxygenation FMT Fosfomycin-Trometamol 3MRGN Three-multiresistant Gram-negative MRI Magnetic resonance imaging ICU Intensive care unit TrBx Transrectal prostate biopsy

* Marked cases were reclassified as non-cases in the respective sensitivity analysis

**Table 3:** Univariable and multivariable logistic regression to estimate the risk of infectious complications based on antibiotic prophylaxis

	Univariable analysis			Multivariable analysis^2^		
Characteristic	OR^1^	95% CI^1^	p-value	OR^1^	95% CI^1^	p-value
Antibiotic						
Ciprofloxacin	–	–		–	–	
Fosfomycin	2.89	1.35–6.87	0.009	2.99	1.39–7.16	0.008

^1^OR = Odds Ratio, CI = Confidence Interval

^2^ The multivariable analysis included age, biopsy history, prostate volume and diabetes as covariates

### Infectious complications by antibiotic prophylaxis

In total, 38 patients (4.2%) had infectious complications within 4 weeks after TrBx, of whom 12/491 (2.4%) had antibiotic prophylaxis with CIP and 26/422 (6.2%) with FMT. The mean latency to the clinical presentation with a suspected infectious complications was 4.5 days (SD 3.9). Most patients with infectious complications presented with fever (84.2%) or specific urological symptoms (68.4%) (Table. 2).

According to the univariable and multivariable logistic regression results, patients who received FMT had 2.99 (95% CI 1.39–7.16, *p* = 0.008) greater odds for infectious complications than compared to those in the CIP group (Table 3).

To assess the robustness of our findings, we conducted three sensitivity analyses. First, we identified 6 patients (marked in Table 2) who presented with infections characterized by nonspecific symptoms or who experienced prolonged latency before symptom onset. After reclassifying these patients as non-cases, refitting the primary multivariable logistic regression model yielded essentially unchanged results (OR 2.69, 95% CI 1.18–6.88, *p* = 0.026) (Supplementary Table 2).

Second, in a subset of 704 patients, we added the total number of biopsy cores as a covariate in the multivariable analysis. The median number of total biopsy cores was 19 (IQR 17–22) in patients without infectious complications and 20 (IQR 18–23) in patients with infectious complications. The addition of the total biopsy count as an independent predictor did not significantly improve the performance of the multivariable model and was therefore omitted from the final model.

Third, to explore potential confounding by informed resistance screening, we compared patients with and without baseline screening. Although screened patients had a higher proportion of complications (CIP: 3.9% vs. 2.1%; FMT: 8.0% vs. 5.7%), adding baseline resistance screening as a covariate did not significantly improve the performance of the multivariable model and was likewise omitted from the final model (Supplementary Table 3).

### Infectious complications requiring hospitalization

Grading and a detailed description of infectious complications according to the Clavien–Dindo classification (CDC) are presented in Table 2 and Supplementary Table 4. Infectious complications ≥ grade 2 required hospitalization and intravenous antibiotic treatment in 22/38 (57.9%) patients, 8/12 (66.6%) in the CIP arm and 14/26 (53.5%) in the FMT arm (*p* = 0.7). The mean duration of hospitalization was 4.7 days (SD 2.2) in the overall cohort, 5.9 days (SD 2.5) in the CIP group compared to 4.0 days (SD 1.7) in the FMT group.

Notably, infectious complications ≥ grade 3 occurred in 3/12 patients (25%) in the CIP arm and 5/26 patients (18.8%) in the FMT arm, with one grade 5 complication due to fulminant sepsis in the FMT arm.

### Antibiotic resistance

Patients at risk for resistant *E. coli* were screened at baseline with rectal swabs, and the rates of screenings performed before biopsy by assigned antibiotic prophylaxis were similar in the CIP group (24.4%) and FMT group (22.0%). The rectal swabs revealed antibiotic-resistant *E. coli* in 28/219 (12.8%) of all screened patients, 22/125 (17.6%) of whom would have received CIP and 6/94 (6.4%) of whom would have received FMT. The overall detection rate of resistant *E. coli* at our centre was consistent during the observation period, with a mean resistance rate to CIP of 20.9% (SD 4.3) and a mean resistance rate to FMT of 1.1% (SD 0.7) (Supplementary Fig. 2).

Microbiological samples (urine and/or blood cultures) were positive in 16/38 patients (42.1%) with infectious complications; 13/38 (34.2%) were negative, and 9/38 (23.6%) had missing data. *E. coli* was identified in 11/16 (68.8%) patients with positive microbiological results, including two cases of multidrug-resistant strains (3MRGN). *K. pneumoniae* was found in 2 patients (12.5%), and *K. oxyctoca* and *B. fragilis gr.* Were each identified in 1 patient (6.3%). In one case (6.3%), both *E. coli* and *E. faecalis* were detected (Table 2).

In the CIP group, none of the identified pathogens were susceptible to the prescribed prophylaxis; only one of six cases showed intermediate susceptibility to CIP at elevated doses. In the FMT group, pathogens were susceptible to prophylaxis in 3/10 cases, resistant in 3/10, and susceptibility was unknown in 4/10 cases (Table 2).

## Discussion

Our study provides a comprehensive analysis of the risk of infectious complications associated with antibiotic prophylaxis with CIP or FMT in patients undergoing TrBx. The findings highlight significant differences in infection rates between the two regimens and offer critical insights for optimizing peri-interventional care for prostate biopsy procedures.

The primary finding of our study was the significantly greater rate of infectious complications in the FMT group compared to the CIP group. The adjusted OR of 2.99 (95% CI 1.39–7.16, *p* = 0.008) for infectious complications in the FMT group underscores the clinical relevance of this difference. This is particularly notable compared with emerging nonantibiotic strategies such as TpBx, which showed no significant difference in infectious complications when compared with unscreened patients (those without rectal cultures or antimicrobial resistance screening) undergoing TrBx, with standard antimicrobial prophylaxis consisting of a combination of oral ciprofloxacin and sulfamethoxazole/trimethoprim or intramuscular ceftriaxone or gentamicin in the prospective randomized ProBE-PC trial (OR: 1.06) [18].

Our result aligns with data from an nRCT by Carignan A. et al., which also indicated an increased risk with FMT, contradicting other nRCTs and RCTs that favoured its use [8–11, 19, 20]. As previously noted, trials favouring FMT have shown significant heterogeneity in study design and outcome definition [11]. Furthermore, the geographical origin of these trials might have biased the results, as these trials favoured FMT in regions with high rates of resistance to CIP [21].

Antimicrobial resistance is an increasing global challenge, and our study corroborates this trend [21, 22]. Rectal swab screening revealed a greater prevalence of resistant *E. coli* in patients who received CIP prophylaxis (17.6%) than in those who received FMT (6.4%). This finding may reflect the historical overuse of fluoroquinolones, contributing to a growing reservoir of resistant strains. Although FMT appeared to have lower resistance rates at baseline, the higher observed infection rates with FMT may indicate that other factors, such as its pharmacokinetics or incomplete coverage of rectal flora, contribute to its inferior performance in preventing postbiopsy infections. The use of alternative antibiotic regimens may help mitigate the development of FQ resistance. A meta-analysis indicated that aminoglycosides (AG), cephalosporins (CS), broad-spectrum penicillins, and carbapenems (CP) were non-inferior to CIP in preventing infections after TrBx. However, these findings were limited by low certainty [5]. Additionally, when discussion alternative antibiotic regimens for the prophylaxis of infectious complications after TrBx, it is essential to consider the mode of administration. Notably, AG and CP can only be administered intravenously, while the bioavailability of oral CS also requires consideration [23]. 

Our results have several implications for clinical practice. While FMT has been proposed as an alternative to fluoroquinolones in response to regulatory changes and growing resistance concerns, our findings caution against its routine use without careful patient selection. Specifically, the elevated baseline resistance rates to CIP highlight the importance of prebiopsy rectal swab screening to tailor antibiotic prophylaxis, particularly in patients with risk factors for multidrug-resistant pathogens [24]. Furthermore, our findings support the need for standardized protocols incorporating emerging alternatives, such as augmented antibiotic regimens or the transperineal approach, to reduce the risk of infectious complications. Recent RCTs, such as PREVENT or ProBE-PC, comparing TrBx with antibiotic prophylaxis to TpBx without antibiotic prophylaxis demonstrated favourable outcomes for TpBx, suggesting that it is an effective antibiotic-sparing strategy that strengthens antibiotic stewardship while maintaining cancer detection rates [18, 25]. Although these trials have several limitations, such as the single-center design of ProBE-PC and the limited follow-up period of PREVENT, which only includes complications occurring within seven days after biopsy, they at least confirmed the noninferiority of TpBx in preventing infectious complications while maintaining cancer detection rates. However, when considering the PREVENT trial, which reported zero infectious complications in the TpBx arm, it is important to note that this favourable outcome was not confirmed in a large population-based study. This study revealed a significantly lower sepsis rate for TpBx than for TrBx (1.03% vs. 1.35%), but there was no significant difference in the mortality rate after biopsy (0.07% vs. 0.1%) [26].

Several limitations of our study warrant consideration. As a single-center retrospective study, our findings may not be generalizable to other institutions with different patient populations, biopsy practices, or local resistance patterns. While we adjusted for key covariates, residual confounding cannot be excluded. The absence of systematic molecular resistance profiling may also limit our understanding of the observed differences in outcomes between FMT and CIP. The relatively small sample size of patients with infectious complications, particularly those requiring hospitalization, may limit the statistical power for subgroup analyses. Although the majority of patients received routine counselling 10–14 days after biopsy, including a review of fever or UTI symptoms, residual underreporting bias cannot be fully excluded.

To align with current changes in guideline recommendations, we have already made several adjustments to our standardized protocol: [1] initiating the transition to TpBx [2], screening all TrBx patients for antibiotic resistance until TpBx is fully integrated, and [3] using CIP as the standard antimicrobial prophylaxis for patients with negative resistance screening.

## Conclusion

Our study demonstrated that FMT is associated with a significantly greater risk of infectious complications compared to CIP in patients undergoing TrBx, raising concerns about its safety as a routine prophylactic agent. These findings highlight the need for individualized prophylaxis strategies informed by local resistance patterns and patient-specific risk factors if TrBx is performed. Future research should focus on multicenter studies to validate our results and explore the true burden of infectious complications after TrBx in larger cohorts. Alternative methods, such as augmented or combination prophylaxis regimens and emerging nonantibiotic strategies, including the transition to transperineal prostate biopsy, should be investigated to mitigate infection risks while addressing the global challenge of antibiotic resistance.

## Electronic supplementary material

Below is the link to the electronic supplementary material.

Supplementary Material 1

## Data Availability

The datasets generated and/or analysed during the current study are not publicly available but are available from the corresponding author upon reasonable request.

## Abbreviations

95% CI: 95% confidence intervals
AG: Aminoglycosides
ANOVA: Analysis of variance
CDC: Clavien‒Dindo-classification
CIP: Ciprofloxacin
CP: Carbapenems
CS: Cephalosporins
EAU: European Association of Urology
DRE: Digital rectal examination
FMT: Fosfomycin-trometamol
FQs: Fluoroquinolones
IQR: Interquartile range
mpMRI: Multiparametric magnetic resonance imaging
nRCT: Nonrandomized controlled trial
OR: Odds ratio
PC: Prostate cancer
PI-RADS: Prostate imaging–reporting and data system
RCT: Randomized controlled trial
SD: Standard deviation
SOFA: Sequential organ failure assessment
TpBx: Transperineal prostate biopsy
TrBx: Transrectal prostate biopsy
TRUS: Transrectal ultrasound
UTI: Urinary tract infection
VIF: Variance inflation factor
