# Supplementary material for: Infectious complications in patients undergoing transrectal prostate-biopsy with ciprofloxacin compared to fosfomycin-trometamol
Source: World J Urol. 2025 Jul 26;43(1):460. doi: 10.1007/s00345-025-05818-8 (PMC12296973; doi:10.1007/s00345-025-05818-8)
Supplement: Supplementary file 1 — Supplementary Material 1 [file 345_2025_5818_MOESM1_ESM.doc]

**Infectious complications in patients undergoing transrectal prostate-biopsy with Ciprofloxacin compared to Fosfomycin-trometamol**

**Running title: Infectious complications after prostate biopsy**

Andreas Banner^1,2^, Sebastian Ubber^3^, Ursula Stoces^1^, Christine Meyer^1^, Stephan Madersbacher^1,3^, Igor Grabovac^2^

^1^ Department of Urology, Klinik Favoriten, Vienna, Austria

^2^ Department of Social and Preventive Medicine, Centre for Public Health, Medical University of Vienna, Austria

^3^ Sigmund-Freud Private University, Vienna, Austria

Correspondence:

Andreas Banner, MD ([andreas.banner@gesundheitsverbund.at](mailto:Stephan.madersbacher@gesundheitsverbund.at))

Table of contents

Page 2 …………………………………………………….. Supplementary Fig. 1

Page 3 …………………………………………………….. Supplementary Fig. 2

Page 3 …………………………………………………….. Supplementary Table 1

Page 4 …………………………………………………….. Supplementary Table 2

Page 4 …………………………………………………….. Supplementary Table 3

Page 4 …………………………………………………….. Supplementary Table 4

Supplementary Fig. 1: CONSORT diagram with patient allocations and exclusions

Assessed for eligibility

(n = 960)

Excluded (n = 47)

- Received different antibiotic prophylaxis (n=41)
- Antibiotic unknown (n=6)

Considered for analysis (n = 913)

Received Ciprofloxacin

(n = 491)

Full biopsy core-count (n = 294)

Received Fosfomycin-Trometamol

(n = 422)

Full biopsy core-count (n = 410)

Full-Set Analysis (n = 422)

Subset Analysis with full core count (n=410)

Full-Set Analysis (n = 491)

Subset Analysis with full core count (n=294)

**Supplementary Fig. 2:** Antibiotic resistance rates over time detected in blood cultures (BC) or urine cultures (UC) for CIP and FMT


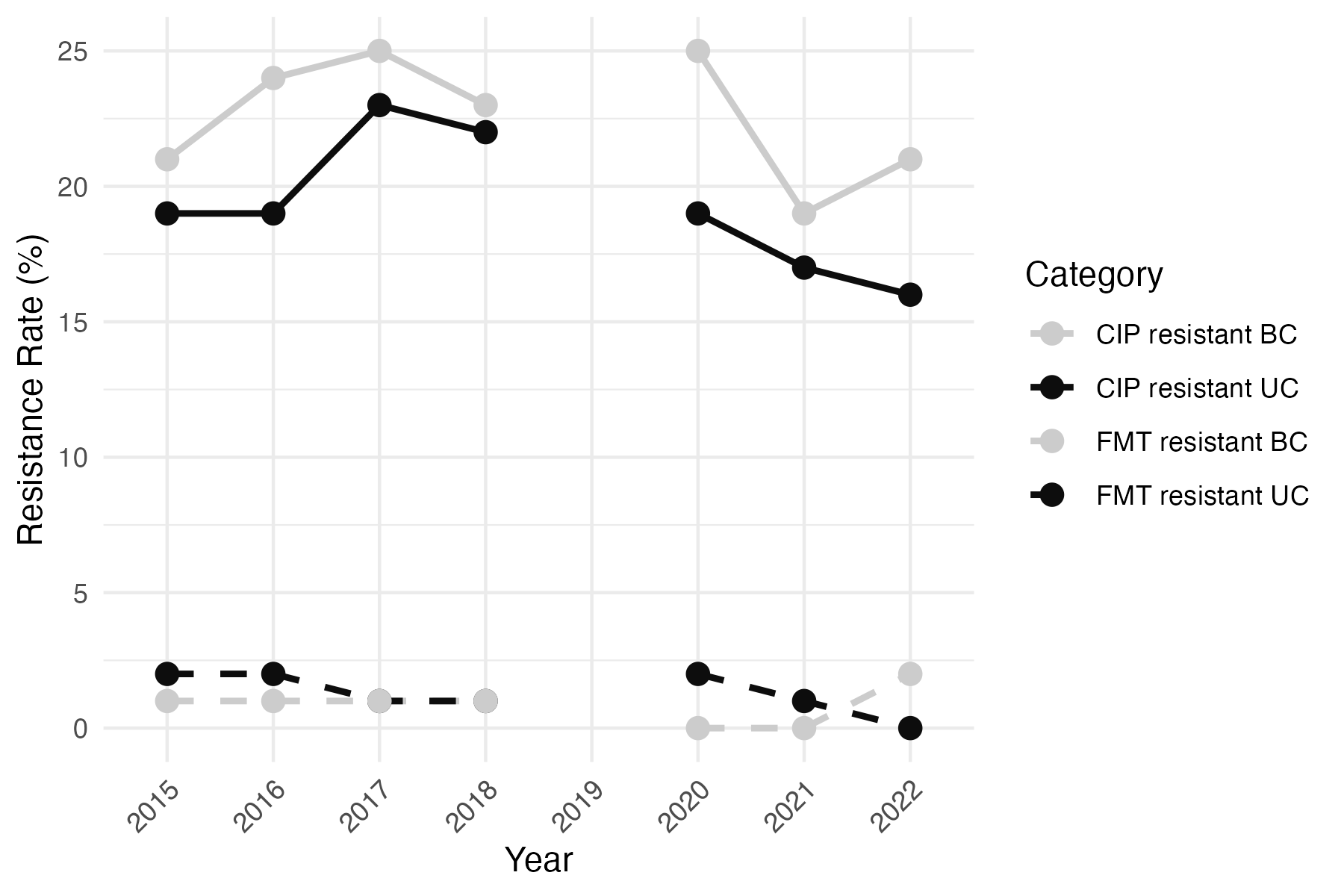


| **Supplementary Table 1:** Clavien-Dindo-Classification of surgical complications (1) | | | |
| --- | --- | --- | --- |
|  | | |  |
| Grade 1 |  | Any deviation from the normal postoperative course without the need for pharmacological treatment or surgical, endoscopic, and radiological interventions. Allowed therapeutic regimens are drugs such as antiemetics, antipyretics, analgesics, diuretics, electrolytes, and physiotherapy | |
| Grade 2 |  | Requiring pharmacological treatment with drugs other than such allowed for grade I complications | |
| Grade 3 |  | Requiring surgical, endoscopic, or radiological intervention | |
| 3a |  | Intervention under local or spinal anaesthesia | |
| 3b |  | Intervention under general anaesthesia | |
| Grade 4 |  | Life-threatening complication requiring medium/intensive care unit management | |
| 4a |  | Single-organ dysfunction | |
| 4b |  | Multiorgan dysfunction | |
| Grade 5 |  | Death of a patient | |

| **Supplementary Table 2:** Sensitivity analysis reclassifying patients with nonspecific symptoms or long latency to symptom onset as non-cases | | | | | | |
| --- | --- | --- | --- | --- | --- | --- |
|  | **Univariable analysis** | | | **Multivariable analysis** | | |
| **Characteristic** | **OR**^1^ | **95% CI**^1^ | **p-value** | **OR**^1^ | **95% CI**^1^ | **p-value** |
| Antibiotic |  |  |  |  |  |  |
| Ciprofloxacin | — | — |  | — | — |  |
| Fosfomycin | 2.62 | 1.16, 6.65 | 0.028 | 2.69 | 1.18, 6.88 | 0.026 |
| ^1^OR = Odds Ratio, CI = Confidence Interval | | | | | | |
| The multivariable analysis included age, biopsy history, prostate volume and diabetes as covariates | | | | | | |

| **Supplementary Table 3:** Proportion of patients with infectious complications, stratified by performance of rectal swabs and antibiotic regimen | | | | | |
| --- | --- | --- | --- | --- | --- |
|  | Infectious complication | **Ciprofloxacin**, N = 491^1^ | | **Fosfomycin**, N = 422^1^ | |
| Rectal swab |  | Yes  (N=103) | No  (N=388) | Yes  (N=88) | No  (N=334) |
|  | Yes | 4 (3.9) | 8 (2.1) | 7 (8.0) | 19 (5.7) |
|  | No | 99 (96.1) | 380 (97.9) | 81 (92.0) | 315 (94.3) |
| ^1^Data are presented as n (%) | | | | | |

| **Supplementary Table 4:** Grading of infectious complications according to the Clavien-Dindo-Classification (CDC), stratified by antibiotic regimen | | |
| --- | --- | --- |
| Characteristic | **Ciprofloxacin**, N = 12^1^ | **Fosfomycin**, N = 26^1^ |
| CDC Grade |  |  |
| 2 | 9 (75%) | 21 (81%) |
| 3a | 3 (25%) | 4 (15%) |
| 5 | 0 (0%) | 1 (3.8%) |
| ^1^Data are presented as n (%) | | |
|  | | |

1. Dindo D, Demartines N, Clavien PA. Classification of Surgical Complications: A New Proposal With Evaluation in a Cohort of 6336 Patients and Results of a Survey. Ann Surg. 2004 Aug;240(2):205–13.
